# Molecular Characterization and Genetic Diversity of Haplogroup E Human Lice in Guinea, West Africa

**DOI:** 10.3390/microorganisms9020257

**Published:** 2021-01-27

**Authors:** Alissa Hammoud, Meriem Louni, Mamadou Cellou Baldé, Abdoul Habib Beavogui, Philippe Gautret, Didier Raoult, Florence Fenollar, Dorothée Misse, Oleg Mediannikov

**Affiliations:** 1 Aix Marseille University, IRD, APHM, MEPHI, IHU-Méditerranée Infection, 13005 Marseille, France; alissa.h.hammoud@gmail.com (A.H.); didier.raoult@gmail.com (D.R.); 2 Aix Marseille University, IRD, APHM, VITROME, IHU-Méditerranée Infection, 13005 Marseille, France; philippe.gautret@club-internet.fr (P.G.); florence.fenollar@univ-amu.fr (F.F.); 3 Faculty of Science, M’Hamed Bougara Boumerdes University, Boumerdès 35000, Algeria; 4 Guinea Institute for Applied Biology Research (IRBAG), 100 BP 75 Kindia, Guinea; cbalde54@gmail.com; 5 Mafèrinyah Rural Health Research and Training Center, 001 B.P. 2649 Conakry, Guinea; beavoguia_h@yahoo.com; 6 Faculty of Medicine, Pharmacy and Odontostomatology, Gamal Abdel Nasser University of Conakry, 001 Conakry, Guinea; 7 MIVEGEC, Univ. Montpellier, IRD, CNRS, 34394 Montpellier, France; dorothee.misse@ird.fr

**Keywords:** head lice, haplogroup E, PHUM540560 gene, *Acinetobacter haemolyticus*, *Acinetobacter* spp., Guinea

## Abstract

*Pediculus humanus capitis*, the head louse, is an obligate blood-sucking ectoparasite that occurs in six divergent mitochondrial clades (A, D, B, F, C and E). Several studies reported the presence of different pathogenic agents in head lice specimens collected worldwide. These findings suggest that head louse could be a dangerous vector and a serious public health problem. Herein, we aimed to study the mitochondrial genetic diversity, the PHUM540560 gene polymorphisms profile of head lice collected in Guinea, as well as to screen for their associated pathogens. In 2018, a total of 155 head lice were collected from 49 individuals at the Medicals Centers of rural (Maférinyah village) and urban (Kindia city) areas, in Guinea. Specimens were subjected to a genetic analysis and pathogens screening using molecular tools. Results showed that all head lice belonged to eight haplotypes in the E haplogroup, with six newly identified for the first time. The study of the PHUM540560 gene polymorphisms of our clade E-head lice revealed that 82.5% exhibited the same polymorphism profile as the previously reported clade A-body lice. Screening for targeted pathogens revealed the presence of *Acinetobacter* spp., while sequencing highlighted the presence of several species, including *Acinetobacter baumannii*, *Acinetobacter nosocomialis, Acinetobacter variabilis, Acinetobacter towneri* and for the first time *Acinetobacter haemolyticus*. Our study is the first to report the existence of the Guinean haplogroup E, the PHUM540560 gene polymorphism profile as well as the presence of *Acinetobacter* species in head lice collected from Guinea.

## 1. Introduction

Sucking lice (*Phthiraptera: Anoplura*) are obligate ectoparasites that have co-speciated with their hosts for at least 77 million years [1]. Humans are colonized by two genera of lice, *Pediculus* and *Phtirus,* which have been strictly feeding on human blood. Nearly 5.6 million years ago the *Phtirus* and *Pediculus* genera diverged along with the divergence of humans and chimpanzee [2]. *Pediculus* genus includes human (*Homo sapiens*) lice, *P. humanus*; chimpanzees (*Pan troglodytes*) lice *P. schaeffi*; and New World monkeys (*Platyrrhini*) lice, *P. mjobergi* [3]. Meanwhile *Phtirus* genus includes *Phtirus pubis,* parasite of humans and *Pt. gorilla,* parasite of gorillas (*Gorilla*) [2,4]. The *P. humanus* includes two ecotypes and is a great public health concern: the *P. humanus corporis* (body louse), which lives in clothing and infests people living in poverty and lack of hygiene. *P. humanus capitis* (head louse), lives in the scalp area, with a worldwide distribution regardless of hygienic conditions [5]. Aside from their role as pests, human lice can be a significant health hazard [3]. Indeed, body lice are the main vector of various serious human pathogens, including *Rickettsia prowazekii*, *Bartonella quintana*, *Borrelia recurrentis* and probably *Yersinia pestis*. These agents respectively cause epidemic typhus, trench fever, relapsing fever and plague [3,6]. Although it is assumed that body lice are a more potent vector for pathogens, the role of head lice as pathogens-vector is still debated and misunderstood [7]. This can be explained by the fact that head lice proceed to a rapid elimination of ingested bacteria, caused by a stronger immune response and therefore a weaker vectorial capacity compared to body lice [8,9]. However, under experimental conditions, the acquisition and the maintenance of *R. prowazekii* and *B. quintana* are reported in head lice [7,10]. Moreover, epidemiological studies have strongly implicated head lice as a vector of infectious pathogens under favorable epidemiological conditions [11]. Various studies have reported that head lice collected worldwide show the presence of DNA from several pathogenic bacteria including: *B. quintana*, *B. recurrentis*, *Borrelia theileri, Y. pestis*, *Coxiella burnetii*, *Rickettsia aeschlimannii*, *Acinetobacter* spp., as well as potential new species of the genera *Moraxella, Psychrobacter*, *Ehrlichia* and *Anaplasma* [12,13,14,15,16,17,18,19,20,21,22,23,24,25,26,27,28,29,30]. These data highlight the fact that head lice can harbor pathogenic bacteria. It is therefore important to prevent epidemics related to lice infestation, thus preventing louse-borne diseases.

Robust phylogenetic studies of human lice based on mitochondrial DNA, mainly *cytochrome b* [*cytb*] and *cytochrome oxidase subunit 1* [*cox1*] genes, have inferred *P. humanus* into six divergent mitochondrial clades (haplogroups): A, D, C, E, B and F, each with distinct geographical distribution [5,14,31,32,33]. Human lice also present an intra-clade diversity in addition to their inter-clade diversity, which is illustrated by several distinct haplotypes for each haplogroup [21,31,34]. Unlike body lice, which only belong to clades A and D, head lice encompass all the genetic diverse clades [18]. Clade A has a global continental distribution and it is the most prevalent [31,34], while clade D is restricted to sub-Saharan African countries, and has so far been reported in the Democratic Republic of Congo (DRC), the Republic of Congo (Congo-Brazzaville), Ethiopia and Zimbabwe [14,21,31]. Clade C has been identified mainly in African and Asian countries, including Ethiopia, Republic of Congo, Nepal, Pakistan and Thailand [18,21,24]. The sister group of clade C, the clade E, has a specific distribution to West Africa where it has been reported with a high prevalence of lice in Senegal, Mali [18], in the Nigerian migrant refugees communities in Algeria [26] and from migrant communities living in Bobigny, France [25]. Recently, two studies reported for the first time the presence of clade E in Central Africa. A new haplotype that has never been described in clade E has been isolated in Congo. While the Malian clade E haplotype, was highly detected in head lice from Gabon [28,29]. Clade B is found in a high diversification in America, it has been reported in Western Europe, Australia, North Algeria, South Africa and Saudi Arabia, and is also present among the remains of head lice from the Roman period dating back to about 2000 years [31,34,35,36,37]. Recently, a novel clade F, the sister group of clade B, was described in French Guiana, in head lice recovered from the Wayampi community living in a remote Trois-Sauts village. This clade was also found in Argentina and Mexico [33]. All these data confirm important facts about the evolutionary history of lice, as well as the ancestors of their human hosts since their migration out of Africa [38].

Despite their clade diversification and ecological niches, The *P. humanus* ecotypes are morphologically and biologically almost similar [3,5]. Previous genetic studies targeting intergenic spacers, using a highly polymorphic markers were not able to differentiate between body and head lice [38,39]. Moreover, a study based on the comparison of head and body lice transcriptomes, reported that the two ecotypes had a single 752-base pair (bp) difference in the Phum_PHUM540560 gene, with differential expression that encodes a hypothetical 69-amino acids protein of unknown function [40]. The PHUM540560 gene and 13 others were thought to be missing in head louse. However, a study conducted by Drali and collaborators [41], showed that the head louse also harbors this gene, but with a rearranged sequence compared to body louse. The variation of the Phum_PHUM540560 gene within the two ecotypes allowed the design and development of a novel molecular tool based on multiplex real-time PCR assays, in order to differentiate the clade A body and head lice.

In Guinea, West Africa, human lice infestation is very frequent but prevalence is never investigated. In this study, we aimed to identify in Guinea, the genetic diversity status of head lice collected from two sites: rural (Maférinyah village) and urban (Kindia city), the Phum_PHUM540560 gene polymorphisms, as well as to assess the occurrence of bacterial pathogens in these lice.

## 2. Materials and Methods

### 2.1. Lice Collection and DNA Extraction

**Figure 1:**
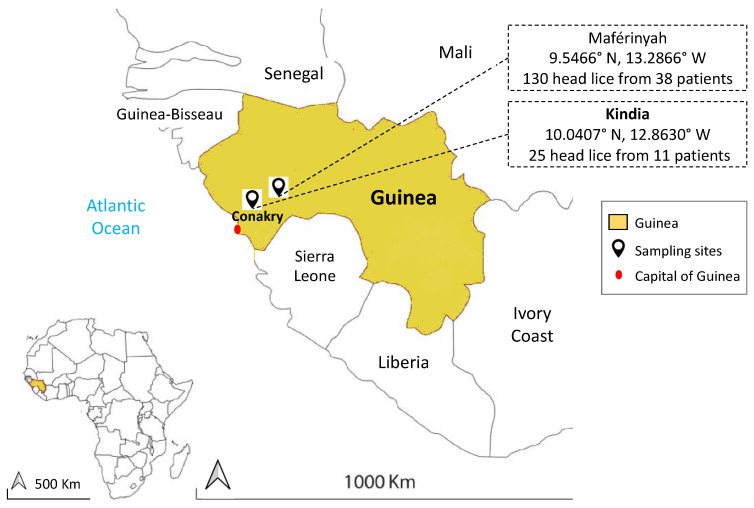
Map of head lice collection sites from infested individuals in two localities in Guinea.

In December 2018, head lice collection was carried out at Medical Centers in two areas: Maférinyah (9.5466° N, 13.2866° W) and Kindia (10.0407° N, 12.8630° W) from Guinea in West Africa (Figure 1). All individuals in medical centers with a head lice infestation were asked to perform a complete self-examination for the presence of head and body lice. The medical center personnel obtained verbal consent from the participants and authorization from the head of the medical center to supervise the collection process.

A total of 155 head lice were obtained from 49 individuals (Mean age: 11 (2–62), 96% female): 130 head lice specimens were collected from 38 individuals in Maférinyah (Mean age: 10 (2–35), 100% female), and 25 head lice samples were collected from 11 individuals (Mean age: 16.2 (6–62); 81.9% female) in Kindia. No body lice were found during the examination. The collected head lice were stored in dry sterile conditions at room temperature and then transported to the laboratory of IHU-Méditerranée Infection, Marseille, France and stored at −20 °C until molecular study. Details about age, sex and development stage of lice are listed in Supplementary Materials (Table S1).

In order to decontaminate the external surface and avoid bacterial contamination, each louse specimen was washed and decontaminated as previously described [42]. Dried louse specimen was cut in half lengthwise; the first half was frozen at −20 °C for subsequent studies. Total DNA was extracted from the remaining half using a DNA extraction kit, QIAamp Tissue Kit (Qiagen, Courtaboeuf, France) in the EZ1 apparatus following the manufacturer’s instructions.

### 2.2. Genotypic Status of Lice

#### 2.2.1. Identification of Louse Mitochondrial Haplogroup by qPCR Assays

In order to identify the mitochondrial clades of the lice collected in this study, all DNA samples were analyzed by clade-specific quantitative duplex real time PCR (qPCR) assays targeting a portion of the *cytb* gene. Each duplex is specific to clades A-D and B-C as previously described, noting that the B-C duplex amplifies specimens belonging to clade E, classified as a sub-clade within clade C [18,21]. Clades PCR identification was carried out using a CFX96 Real-Time system (Bio-Rad Laboratories, Foster City, CA, USA). The amplification of DNA was performed using the following parameters: one step of incubation at 50 °C for 2 min (for UDG activation), one step of 95 °C for 5 min for initial denaturation and 45 cycles of 5 s at 95 °C and 30 s at 60 °C. The final reaction volume of 20 μL contained 10 μL of Eurogentec™ Probe PCR Master Mix (Eurogentec, Liege, Belgium), 0.5 μM of each primer, probe and water. To validate the qPCR run, lice with known clades were used as positive controls and master mixtures as negative controls for each test.

#### 2.2.2. Identification of Louse Haplotype by Conventional PCR Assays and Sequencing

In order to perform a phylogenetic study, all head lice specimens were subjected to standard PCR targeting a 347-bp fragment of the *cytb* gene as previously described [38]. PCR amplification was performed in a Peltier PTC-200 model thermal cycler (MJ Research Inc, Watertown, MA, USA). The final volume PCR consisted of a 25 μL, including 12.5 μL Amplitaq gold master mixes, 0.5 μM of each primer, 5 μL DNA template, and water. The thermal cycling profile comprised an incubation step at 95 °C for 15 min, 40 cycles of 1 min each at 95 °C, 30 s at 56 °C and 1 min at 72 °C, followed by a final extension step of 5 min at 72 °C. The results of the amplification were then confirmed by electrophoresis in agarose gel. To proceed with the purification of the PCR products, a NucleoFast 96 PCR plates (Macherey-Nagel EURL, Hoerdt, France) were used according to the manufacturer’s instructions. The amplicons were then sequenced using the Big Dye Terminator Cycle Sequencing Kit (Perkin Elmer Applied Biosystems, Foster City, CA, USA) with an ABI automated sequencer (Applied Biosystems). The electropherograms obtained were then assembled and edited using the ChromasPro software (ChromasPro 1.7, Technelysium Pty Ltd., Tewantin, Australia) and compared with those available in GenBank database by NCBI BLAST (http://blast.ncbi.nlm.nih.gov/Blast.cgi).

#### 2.2.3. Sequences and Phylogenetic Diversity Analysis

In order to assess clade diversity in human lice, the total Guinean head lice-*cytb* sequences obtained in this study were combined and compared with the worldwide *P. humanus-cytb* mitochondrial sequences previously reported (Supplementary Data: Table S2). ClustalW alignments were performed using MEGA software version 6.06. Thereafter, the Maximum-likelihood (ML) analysis was also performed in MEGA6 using the Kimura 2-parameter model for nucleotide sequences under 1000 bootstrap replicates [43]. The *cytb* from *P. schaeffi* (AY696067) was used as an outgroup. A median-joining (MJ) network was also constructed in order to investigate the possible relationships between the haplotypes, using the method of Bandelt performed with NETWORK10.0.0 program (www.fluxus-engineering.com/sharenet.html) [44].

### 2.3. Molecular Investigation of Lice Ecotype

#### 2.3.1. Louse Ecotype Investigation by Multiplex qPCR Assays

In this study, all lice specimens were collected from the scalp region of each individual; however, we were curious to further study our lice ecotypes. Therefore, all lice samples were analyzed by multiplex real-time PCR, targeting a portion of the PHUM540560 gene. This assay was developed to discriminate between body lice and head lice belonging to clade A [41], which has always been known as a worldwide clade that encompass the two ecotypes [31,34]. This assay had never been used to study lice belonging to other clades. We used a clade A head and body louse as positive controls.

#### 2.3.2. Louse Ecotype Investigation by Conventional PCR Assays and Sequencing

To analyze the sequences of the PHUM540560 gene of the Guinean human lice, 40 specimens were randomly selected. Additionally, clade A-human lice were randomly selected from our lice collection, including 7 specimens of Orlando strain from our rabbit rearing colony and 11 Algerian body lice collected from homeless people [45]. These samples were then subjected to standard PCR and sequencing of the target PHUM540560 gene. The sequences obtained were aligned with the specific PHUM540560 sequences of the clade A-body and head lice, described thorough the previous study [41]. Alignment was performed using the BioEdit v 7.0.5.3 software (available online: http://en.bio-soft.net/format/BioEdit.html), in order to reveal the rearranged sequences of the head lice PHUM540560 gene collected in Guinea compared with those of the clade A-human lice.

### 2.4. Screening for the Presence of Pathogen’s DNA

#### 2.4.1. Identification of Pathogen’s DNA by qPCR Assays

In order to screen louse-borne pathogens, a qPCR was performed for all head lice samples targeting the presence of various pathogenic bacteria: *Rickettsia* spp., *Borrelia* spp., *B. quintana, Y. pestis, C. burnetii*, *Anaplasma* spp. and *Acinetobacter* spp., using specific primers and probes, as previously reported (Table 1).

Initially, we pooled our DNA’s template into 8 samples of 10 μL/specimen for each pool; each sample was in a proportion of 1 Log of its initial concentration. Modifications of the qPCR cycles threshold (Ct) from 40 Ct to 45 Ct were performed to ensure that no potentially positive samples were missing from each pool. Thereafter, each louse from each positive pool with a Ct ≤ 38, was tested individually. The qPCR analysis was performed as described above for the *cytb*, a positive control of the targeted DNA and a negative control of master mixtures were included in each PCR run. Samples positive for *Acinetobacter* spp. were subjected to qPCR specific for *Acinetobacter baumannii*, targeting the *OmpA*/*MotB* gene, as previously reported (Table 1).

#### 2.4.2. Identification of Pathogen’s DNA by Conventional PCR and Sequencing

In order to identify the species of *Acinetobacter*, positive samples from qPCR were subjected to a standard PCR targeting a portion of the *rpoB* gene (zone1) using the primers and conditions previously described (Table 1). Successful amplification was confirmed by electrophoresis via agarose gel, amplicons were prepared and sequenced using similar methods as described above. Due to a low specificity of above-mentioned primers, a new specific PCR system was designed in this study in order to amplify only and specifically the DNA of *Acinetobacter* spp. targeting a different portion of the same *ropB* gene. The Fasta-file was constructed from the sequences available in the GenBank database for all *Acinetobacter* species as well as for *Moraxella* spp. and *Psychrobacter* spp. Sequences were aligned using BioEdit v 7.0.5.3 software (available online: http://en.bio-soft.net/format/BioEdit.html) to reveal conserved areas as target regions for *Acinetobacter* spp. specific primers. This region was submitted in Primer3 software v. 0.4.0 (available online: http://primer3.ut.ee/) to determine candidate primers based on the criteria for the primer design. PCR primers settings were in accordance with the guidelines as previously described and as recommended by Invitrogen^TM^ (available online: https://www.thermofisher.com/fr/fr/home/brands/invitrogen.html) and Applied Biosystems^TM^ (available online: https://www.thermofisher.com/fr/fr/home/brands/applied-biosystems.html). Then, the melting temperatures of the primers were tested using the free online software Oligo Analyzer 3.1 (available online: https://eu.idtdna.com/calc/analyzer). Primers were then synthesized by Eurogentec (Liège, Belgium). PCR systems and their target gene are described in Table 1.

### 2.5. Acinetobacter Resistance to Carbapenem

To eventually investigate this paradigm in our study, all positive *A. baumannii*-Guinean head lice were subjected to qPCR analyzes targeted three carbapenem resistant encoding genes including *bla_OXA-23_*, *bla_OXA-24_* and *bla_OXA-58_*, as previously described (Table 1). Results were considered positive when the cycle threshold value of real-time PCR is ≤35.

## 3. Results

**Figure 2:**
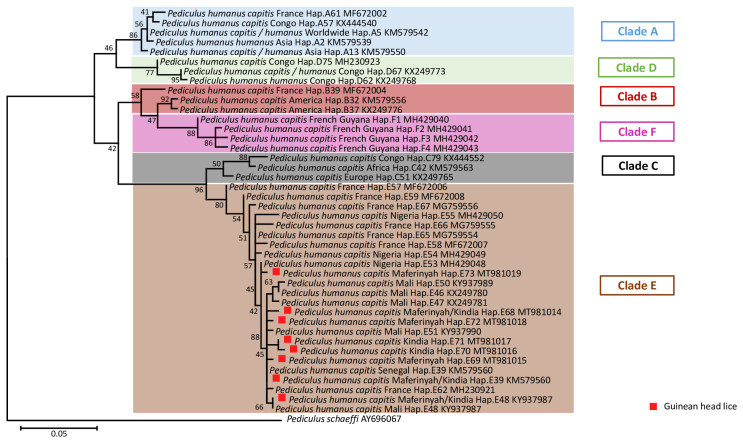
Maximum-likelihood (ML) phylogenetic tree of the mitochondrial *cytb* gene showing the relationship of haplotypes identified in this study with other *P. humanus* haplotypes reported in the literature. Phylogenetic inference was conducted in MEGA 7 using the maximum likelihood method under the Kimura 2-parameter with 1000 bootstrap replicates. There were a total of 141 positions in the final dataset.

**Figure 3:**
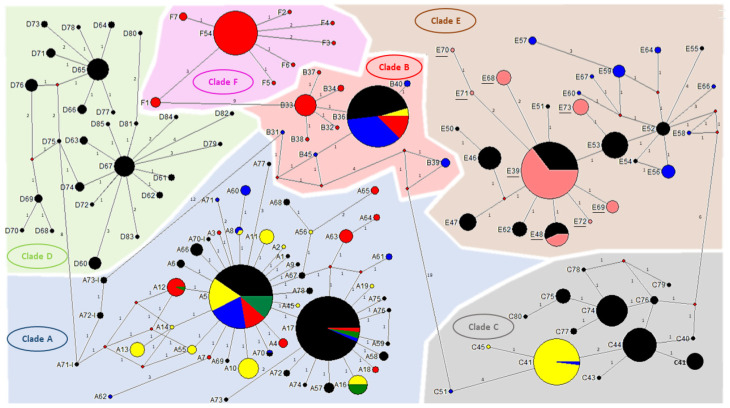
*Cytb* haplotype networks of human body and head lice including our samples. Each circle indicates a unique haplotype, and variations in circle size are proportional to haplotype frequencies. Pie colors and sizes in circles represent the continents and the number of their sequence for a haplotype. The length of the links between nodes is proportional to the number of mutations. The types of haplotypes identified in this study are underlined. Besides, all *P. humanus* lice were tested by multiplex qPCR targeting the PHUM540560 gene to investigate their ecotype; this method was used previously to distinguish between head and body lice belonging to clade A [41]. All our specimens were collected by the patients from their scalp hair, and belong to clade E. Using the multiplex qPCR method; all the 155 Guinean head lice specimens exhibited a FAM-labeled probe amplification specific to the body lice profile. These results confirm the fact that the PHUM540560- multiplex qPCR is a restricted molecular tool to differentiate only between clade A-human lice. Based on these data, we proceeded with the analysis of the PHUM540560 gene sequences of our samples and those belonging to clade A reported in the literature [41]. For this purpose, diverse human lice specimens encompassing both ecotypes were randomly selected from our lice collection. These samples were then subjected to standard PCR and sequencing of the targeted PHUM540560 gene. The obtained sequences were aligned with sequences of body and head lice. Alignment was conducted using the BioEdit v 7.0.5.3 software (available online: http://en.bio-soft.net/format/BioEdit.html). The rearranged sequences of the clade E Guinean-head lice PHUM540560 gene were revealed and to compared to the polymorphisms of the clade A body and head lice.

This study included 155 head lice specimens collected from 49 individuals, (96% female and 4% male), from two regions of Guinea, Maférinyah and Kindia. First, All *P. h. capitis* specimens were subjected to a duplex qPCR to determine their clade. The results of the amplification curve revealed that our samples were positive for the clade C–E. Standard PCR and sequencing showed that all our samples belonged to the haplogroup E. For the phylogenetic analysis, we were able to generate 141 sequences from the 155 samples analyzed, due to the low DNA concentration. The generated Guinean sequences were then aligned and combined with all sequences available for *cytb* haplotypes and were then used to construct a maximum-likelihood (ML) tree (Figure 2) and a median-joining (MJ) network (Figure 3). The results revealed the existence of 8 haplotypes, including 6 new haplotypes referred here as E68, E69, E70, E71, E72 and E73 with the attributed GenBank accession numbers MT981014-MT981019 respectively. In addition, E39 and E48, the two most prevalent haplotypes present in clade E, accounted for the majority of our lice samples. Indeed, most of our head lice specimens, 96 (68.1%), belonged to the haplotype E39, 12 (8.5%) to the haplotype E48 and 33 (23.4%) to the six novel haplotypes, with 10 (30.3%) to E68, 8 (24.3%) to E69, 1 (3%) to E70, 1 (3%) to E71, 1 (3%) to E72 and 12 (36.4%) to E73.

**Figure 4:**
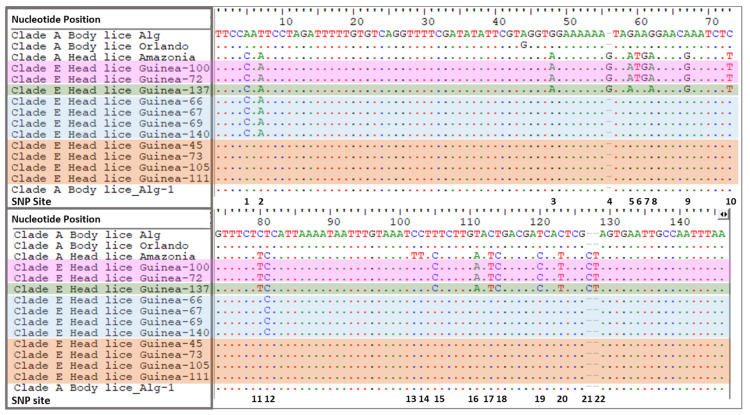
Alignments of a portion of Guinean head lice-Phum_PHUM540560 gene sequences with those from body and head lice reported in this study and previously in the literature. HL Amazonia [40] represent the 22 SNPs that are specific to clade A-head lice. BL Algeria (Alg) and Orlando represent the PHUM540560 gene profile specific to clade A-body lice. HL Guinea group contains 11 specimens: Orange block nucleotide represent 0 SNPs specific to head lice, blue block nucleotides represent 3 SNPs specific to head lice; green block nucleotides represent 18 SNPs specific to head lice and pink block nucleotides represents 20 SNPs that are specific to *P.h. capitis.* BL: body louse; HL: head louse; *P.h.corporis* strain USDA 1103172108290 Phum_PHUM540560 (gene sequence available in GenBank accession N. NW_002987859.1).

The comparison of the PHUM540560 gene of Clade A-head lice and clade A-body lice revealed the presence of 22 single nucleotides polymorphisms (SNPs) on the head lice-PHUM540560 gene sequence. The first two point mutations are situated on the first exon and the remainder of the polymorphisms are spread throughout the first intron [41]. Interestingly, none of our clade E-head lice showed the clade A head lice-PHUM540560 gene profile. Indeed, 33/40 (82.5%) of Guinean head lice are characterized by the absence of all SNPs present in clade A- head lice, thus exhibiting a clade A-body lice profile. Meanwhile, 7/40 (17.5%) of our samples displayed different head lice SNPs: 4/7 (57.1%) revealed the existence of 3 SNPs, 1/7 (14.3%) the existence of 18 SNPs and 2/7 (28.6%) the existence of 20 SNPs. Regarding the SNPs profile of our tested body lice, all (18 samples) had the same PHUM540560 gene profile of clade A-body lice by having 0 SNPs reported by Drali et al. [41]. Details of PHUM540560 sequences alignments are presented in Figure 4. Details of head lice numbers, codes and PHUM540560 gene SNPs profiles are listed in Table 2.

**Figure 5:**
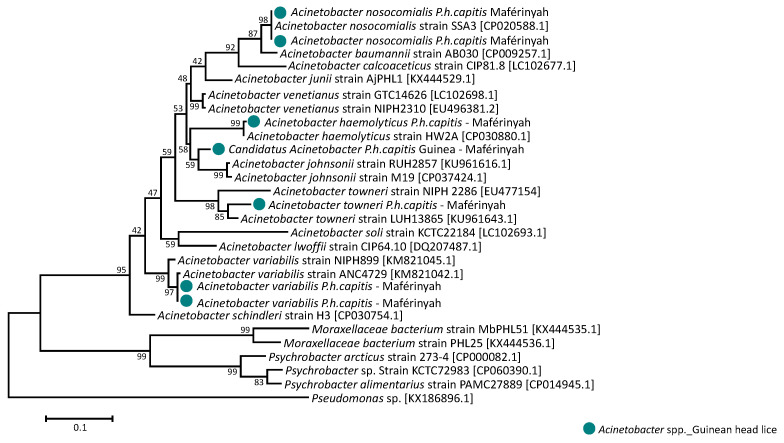
Phylogenetic tree highlighting the position of the *Acinetobacter* species identified in the head lice collected from Guinea compared to *Acinetobacter* spp. available in the GenBank database. Phylogenetic inferences were conducted in MEGA 7 using the maximum likelihood method based on the Kimura 2-parameter model for nucleotide sequences. Statistical support for internal branches of the tree was evaluated by bootstrapping with 1000 replicates. There was a total of 7 positions in the final dataset.

In addition, none of the head lice samples revealed the presence of *Rickettsia* spp., *B. quintana*, *Borrelia* spp., *Y. pestis*, *C. burnetii* and *Anaplasma* spp. However, the DNA of *Acinetobacter* spp. was found in 14/155 (9%) samples collected from 11/46 (24.5%) individuals. All positive samples were collected in 14/130 (10.8%) Maférinyah. Positive *Acinetobacter* specimens were then tested for a specific *A. baumannii*-qPCR, revealing that 2 of the 14 (14.3%) samples were positive, these samples belonged to the haplotype E39 and were collected from 2 different patients in Maférinyah. The first amplification of *Acinetobacter* DNA in positive head lice samples showed the presence of the DNA from *Moraxella bacterium* and *Psychrobacter* spp., which explains the low specificity of the target gene-amplification by these primers. We designed a more specific system targeting the *rpoB* gene of this bacterium and, due to the low concentration of *Acinetobacter* DNA in our positive head lice, we succeeded in amplifying the DNA of the pathogen in only 7/14 positive samples. Sequencing and blast analysis of 350-bps fragment *rpoB* gene revealed that 6/7 (85.7%) of our sequences shared 99–100% identity with 4 *Acinetobacter* species including: 2/7 (28.6%) E69 and E39 head lice matching with *Acinetobacter nosocomialis,* 2/7 (28.6%) E39 and E48 head lice with *Acinetobacter variabilis,* 1/7 (14.2%) E69 head louse with *Acinetobacter towneri* and finally, 1/7 (14.2%) E48 specimen with *Acinetobacter haemolyticus*. The remaining generated sequence 1/7 (14.3%), shared a lower similarity (<94%, coverage 100%) with two *Acinetobacter* species: *A. johnsonii* and *A. venetianus*, suggesting that this *Acinetobacter* is a potential new specie, named here “Candidatus *Acinetobacter P.h capitis* Guinea”. This positive sample belonged to haplotype E39. All positives head lice for *Acinetobacter* species were collected at Maférinyah, and none at Kindia. Interestingly, one of the patients included in this study was infested with four head lice, two of them were infected with *A. nosocomialis* and *A. variabilis;* belonging respectively to haplotypes E48 and E39. The remaining seven of the 14 sequences (50%) also had similarities with *Acinetobacter* spp. However, the sequences were of poor quality, which is assumed to be due to the co-infection of several *Acinetobacter* species. The phylogenetic tree of all *Acinetobacter* species identified in this study is presented in Figure 5. Details about alignment and the sequences of a 288-bp *rpoB* gene fragment of the detected *Acinetobacter* species presented in supplementary Data Figure S1

In our study, none of the DNA *A. baumannii* samples tested positive for carbapenem’s-resistant encoding genes (*bla_OXA-21_, bla_OXA-24_, bla_OXA-58_*).

## 4. Discussion

The present study is the first to investigate both the phylogeny and associated pathogens of head lice collected in Guinea. A total of 155 head lice were collected from 47/49 (95.9) females and 2/49 (4.1%) males living in two different rural and urban areas, Maférinyah a village, and the city of Kindia, Guinea, West Africa. A genetic study using the qPCR duplex first showed that all the lice samples analyzed belonged to the mitochondrial clade C or E. Standard PCR and sequencing revealed that all the head lice belonged to the haplogroup E. The qPCR duplex method is not discriminative enough for the screening of African human lice, which mainly belong to haplogroups C and E. A specific clade E monoplex system was previously designed but not used in our study [18]. For further studies, a more specific duplex system will be optimized, for better identification of African human lice, as well as to establish an optimal qPCR duplex for the discrimination of all human lice belonging to all existing haplogroups, including the recently described clade F. The phylogenetic study showed the existence of 8 haplotypes including 6 novels described for the first time in this study. The presence of clade E in both rural and urban communities in Guinean lice is not surprising, as it confirms the high prevalence of the “African endemic” clade E, as previously reported so far [18,26,28,29]. The most prevalent haplotype reported in our head lice is the E39 obtained with 68.1%, followed by the haplotype E48 with 8.5% of the total prevalence. In addition, six new haplotypes were identified in Guinean head lice, including E68 (7.1%), E69 (5.6%), E70 (0.71%), E71 (0.71%), E72 (0.71%) and E73 (8.5%). So far, haplogroup E has only been found in head lice collected in West African countries, including Senegal and Mali [18], in head lice samples collected from Nigerians refugees in Algeria [26] and in *P.h. capitis* collected from migrant communities living in Bobigny, France [25]. However, a recent study showed for the first time the presence of clade E, more specifically the E62 haplotype in Central Africa, in Congo, suggesting that Congonians are in direct contact with West African populations or travelers arriving form West African countries [28]. More recently, this clade has also been found in head lice collected from individuals in Gabon, belonging to haplotype E46 [29], already reported among lice collected in Mali [18]. These results suggest that the significant migratory exchange between Gabon and the Republic of the Congo can be the source for the clade E expansion [29]. In addition, among the 141 head lice *cytb* sequences analyzed, 4 lice with two different haplotypes, E39 and E70, were collected from the same 34-year-old woman. In addition, four haplotypes were also identified within the same person infested with 11 lice, in Kindia, belonging to haplotypes E39, E48, E71 and E73. Co-infestations by different mitochondrial DNA clades of human head lice within the same person were reported previously, showing that human lice belonging to different clades can live in sympatry and interbreed [36,39], including the association of clades A and B [36], clade A and D [14,29], clade A and C [39]. All these results underline the fact that more phylogenetic studies on human lice-mitochondrial clades, from larger sampling zones, with different geographical areas, and more human lice samples, should be carried out to broaden our knowledge of the inter and intra-haplogroup diversity.

In this study, we investigated the Phum540560 gene polymorphisms of Guinean head lice belonging to clade E. We could not confirm the ecotype of our head lice samples using the Phum540560-multiplex qPCR, previously used to discriminate between *P. humanus* ecotypes belonging to clade A, therefore, our results are consistent with those reported previously [41], the clade E head lice ecotype cannot be identified by this molecular tool. However, in our study, we were interested in analyzing the Phum_PHUM540560 gene sequences from Guinean head lice to better understand their polymorphism profile. Interestingly, none of our clade E-head lice showed the clade A head lice-PHUM540560 gene profile. Indeed, 33/40 (82.5%) of Guinean head lice were characterized by the absence of all the above-mentioned SNPs present in clade A-Amazonian head lice, consequently exhibiting a clade A-body lice profile, while 7/40 (17.5%) samples showed different kinds of SNPs: 4/7 (57.1%) revealed the existence of 3 SNPs, 1/7 (14.3%) the existence of 18 SNPs and 2/7 (28.6%) the existence of 20 SNPs. These results highlight the fact that the majority of the Clade E-Guinean head lice exhibit the profile of the clade A-body lice, noting that the clade A lice is the origin of clades B and C, where clade B head lice were reported to diverge from clade A between 0.7 and 1.2 Mya, whereas clade C is even older (ca. 2 Mya) [11]. These results should encourage further study of the polymorphism profile of the PHUM540560 gene from *P. humanus* belonging to the six divergent mitochondrial clades reported so far. In addition, further studies, with a wider sampling, are necessary to study a larger portion of the Phum_PHUM540560 gene. These investigations will allow a better understanding and will probably lead us to design a more efficient molecular tool, which will be able to discriminate between the two ecotypes. At this point, we can affirm the fact that the morphological, biological and genetic characteristics of *P. humanus* species are almost similar and remain obscure. However, body and head lice are extremely different in their ecological niches, which remain, until now, the main criterion for distinguishing between these two ecotypes.

In recent decades, the paradigm that *P.h. corporis* was the only vector of dangerous diseases has been challenged [8]. Indeed, many studies have reported the presence of several pathogenic agents in head lice specimens collected worldwide [12,13,14,15,16,17,18,19,20,21,22,23,24,25,26,27,28,29,30]; thus, demonstrating that the potential pathogen-vector-competence of the head louse is not yet understood [8]. In this study, among all the bacteria examined, we found the presence of *Acinetobacter spp.* in only 14 head lice infesting 11 individuals. Findings from previous studies reported a worldwide spread of several *Acinetobacter* species, including *A*. *baumannii*, *A*. *junii*, *A*. *ursingii*, *A*. *johnsonii*, *A*. *schindleri*, *A*. *lwoffii*, *A*. *nosocomialis*, *A*. *towneri, A*. *variabilis, A. radioresistens, A. calcoaceticus, A. soli, A. pittii* and potential new species in head lice collected from different population categories, including elementary school children in Algeria [26,27], France [22], Thailand and Georgia-USA [19,24], of the Pygmy population in the Republic of Congo [21], Nigerian refugee children in Algeria [26] and, more recently, in head lice collected from healthy women in Gabon [29], and even in ancient Roman-era head lice remains [32]. The diversification of *Acinetobacter* species was reported from head lice belonging to the majority of the existing head lice-haplogroups A, D, C, E and B, and in different haplotypes of each haplogroup [11]. Among the 14 *Acinetobacter* positives *P.h. capitis* samples, we identified the existence of *A. baumannii*, *A. nosocomialis, A. variabilis A. towneri* and for the first time in head lice *A. haemolyticus* as well as one potential new specie named here “Candidatus *Acinetobacter P.h. capitis* Guinea”. Based on these results, we confirmed the diversity of the *Acinetobacter* species in head lice. Our study is the first to describe the presence of *A. haemolyticus* in human head lice. *A. haemolyticus* is a pathogenic bacterium widely distributed in nature and commonly found in soil, water and hospitals. Like *A. baumannii*, *A. johnsonii* and *A. junii*, *A. haemolyticus* is an important clinical microorganism responsible for nosocomial infections and associated with endocarditis, bacteremia and others types of infections in hospitals [54].

Body lice can also be infected by a diverse *Acinetobacter* species, including *A. baumannii, A*. *johnsonii*, *A*. *berezeniae*, *A*. *nosocomialis* and *A*. *variabilis* [23,45]. Indeed, *A*. *baumannii* was isolated for the first time from body lice collected on homeless people in France and, subsequently, the bacterium was detected in body lice collected worldwide [54]. Most of the *Acinetobacter* species are pathogenic bacteria that can survive for a long period in the environment and have been associated with carbapenem resistance, especially *A. baumannii* and *A. haemolyticus,* because of their multiple resistance to many common antibiotics [55].

In recent decades, *Acinetobacter* bacteria have shown high ability to develop resistance to almost all major classes of antibiotics. So far, the incidence of carbapenem resistance in *A. baumannii* has continued to increase worldwide [56]. In human lice, *A. baumannii* isolates were remarkably susceptible to carbapenem. Indeed, a study performed on head lice collected in Senegal reported that 21.4% of the positive *A. baumannii-* head lice harbored a *bla_OXA-23_* carbapenem resistant encoding gene [56]. None of our positive-*A. baumannii* head lice were positive to the carbapenem’s-resistant encoding genes (*bla_OXA-21_, bla_OXA-24_, bla_OXA-58_*). Further studies are needed to investigate the association between *Acinetobacter* infections and human lice, the compatibility of *Acinetobacter* strains present in lice and those responsible for human infections, as well as investigate the carbapenem’s-resistant in *A. baumannii* strain present in human lice.

## 5. Conclusions

Herein, we report the first molecular data on Guinean head lice genetic diversity and their associated pathogens. We confirmed the West African clade E distribution of head lice as well as the *Acinetobacter* species diversification. Head lice is a public health concern, further studies are needed to better understand their genetic diversity and their role as a vector for pathogenic bacteria.

## Figures and Tables

**Table 1:** Real time PCR and conventional PCR primers and probes used in this study.

Target	Name	Primers and Probes (5′-3′)	Source
*P. humanus*	*Cytb*. Duplex A/D	FAM-CATTCTTGTCTACGTTCATATTTGG-TAMRA	[18]
		VIC-TATTCTTGTCTACGTTCATGTTTGA-TAMRA	
		F_GATGTAAATAGAGGGTGGTT	
		R_GAAATTCCTGAAAATCAAAC	
	Cytb. Duplex B/C-E	FAM-GAGCTGGATAGTGATAAGGTTTAT-TAMRA	
		VIC-CTTGCCGTTTATTTTGTTGGGGTTT-TAMRA	
		F_TTAGAGCGMTTRTTTACCC	
		R_AYAAACACACAAAAMCTCCT	
	*Cytb*	F_GAGCGACTGTAATTACTAATC	[38]
		R_CAACAAAATTATCCGGGTCC	
	Phum540560	FAM-CGATCACTCGAGTGAATTGCCA-TAMRA	[41]
		VIC-CTCTTGAATCGACGACCATTCGCT-TAMRA	
		GTCACGTTCGACAAATGTT	
		TTTCTATAACCACGACACGATAAAT	
*Rickettsia* spp. citrate synthase (gltA)	*RKNDO3*	FAM-CTATTATGCTTGCGGCTGTCGGTTC-TAMRA	[46]
		F_GTGAATGAAAGATTACACTATTTAT	
		R_GTATCTTAGCAATCATTCTAATAGC	
*Borrelia* spp. *16S ribosomal RNA*	*Bor16S*	FAM-CCGGCCTGAGAGGGTGAACGG-TAMRA	[47]
		F_AGCCTTTAAAGCTTCGCTTGTAG	
		R_GCCTCCCGTAGGAGTCTGG	
*Bartonella quintana*	*yopP*-Hypothetical intracellular effector	FAM-GCGCGCGCTTGATAAGCGTG-TAMRA	[48]
		F_GATGCCGGGGAAGGTTTTC	
		R_GCCTGGGAGGACTTGAACCT	
*Yersinia pestis*	*PLA*	FAM-TCCCGAAAGGAGTGCGGGTAATAGG-TAMRA	[13]
		F_ATG GAG CTT ATA CCG GAA AC	
		R_GCG ATA CTG GCC TGC AAG	
*Coxiella burnetii*	*IS1111*	FAM- CCGAGTTCGAAACAATGAGGGCTG-TAMRA	[49]
		F_CGCTGACCTACAGAAATATGTCC	
		R_GGGGTAAGTAAATAATACCTTCTGG	
*Anaplasma* spp. 23S ribosomal RNA	*TtAna*	FAM-GGATTAGACCCGAAACCAAG-TAMRA	[50]
		F_TGACAGCGTACCTTTTGCAT	
		R_TGGAGGACCGAACCTGTTAC	
*Acinetobacter* spp. RNA polymerase β subunit gene	*rpoB*	FAM-CGCGAAGATATCGGTCTSCAAGC-TAMR	[22]
		F_TACTCATATACCGAAAAGAAACGG	
		R_GGYTTACCAAGRCTATACTCAAC	
	*rpoB* (zone1)	F_TAYCGYAAAGAYTTGAAAGAAG	[51]
		R_CMACACCYTTGTTMCCRTGA	
	*rpoB* (zone1)	F_TACAARATCTTYGAAGAAGC	This study
		R_CCACAACADAGDTTGTARRA	
*Acinetobacter baumanii.* Type VI secretion system *OmpA*/*MotB*	*OmpA*/*MotB*	FAM_AAGTCGCCAAGAAACCTTGA_TAMRA	[52]
		F_TCAACATCACAATCTTTAGTAGCTGA	
		R_CGCTCTTGCCAGCATAAAGA	
Carbapenems genes	*OXA-23*	6-FAM-CCAGTCTATCAGGAACTTGCGCGA-BHQ_1	[53]
		F_GACACTAGGAGAAGCCATGAAG	
		R_CAGCATTACCGAAACCAATACG	
	*OXA-24*	TET-AGTAACACCCATTCCCCATCCACTTTT-IABkFQ	
		F_GATGACCTTGCACATAACCG	
		R_CAGTCAACCAACCTACCTGTG	
	*OXA-58*	Cy5-TGGACCAATACGACGTGCCAATTCT-IAbRQSp	
		AAGATTTTACTTTGGGCGAAGC	
		CAACTTCCGTGCCTATTTGC	

**Table 2:** PHUM540560 gene SNPs profile of Guinean clade E-head lice, Amazonian and Algerian clade A-head lice

Ecotype	N. of HL	Clade and Origin	N. of SNPs	Present SNPs
HL	1	A-Amazonia	22	SNP 1–22
BL	1	A-Algeria	0	-
HL	1	E-Guinea	18	SNP 1–5, 8–12, 15–22
HL	2	E-Guinea	20	SNP 1–12, 15–22
HL	4	E-Guinea	3	SNP 1, 2 and 12
HL	33	E-Guinea	0	-

N: Number; HL: head lice, BL: body lice SNPs: Single Nucleotide Polymorphisms. A-Amazonia & A-Algeria represent reference sequences for head and body lice.

## Data Availability

Sequences of the 8 haplotypes, including 6 new haplotypes referred here as E68, E69, E70, E71, E72 and E73 with the attributed GenBank accession numbers MT981014-MT981019 respectively of the *cytb* gene of *Pediculus humanus* gene are present on the following link https://www.ncbi.nlm.nih.gov/nuccore/MT981014 https://www.ncbi.nlm.nih.gov/nuccore/MT981019.

## Supplementary Materials

The following are available online at https://www.mdpi.com/2076-2607/9/2/257/s1. Table S1. Table representing details about lice collected in Rural Maferenya and Urban Kindia, Guinea. Table S2. Geographical and frequencies occurrences of *cytb* haplotypes of human head and body lice worldwide. Figure S1. Alignment of a 288-bp fragment of the rpoB gene of *Acinetobacter nosocomialis* strain SSA3 [CP020588.1], *Acinetobacter variabilis* strain ANC 4771 [KM821044.1], *Acinetobacter haemolyticus strain* 11616 [CP032002.1] and *Acinetobacter towneri strain* 205 [P048014.1] with the five *Acinetobacter* species described in this study using BioEdit software. Mutations of our potential new specie ‘*Acinetobacter* sp *P.h.capitis*–Maferenya’ regarding other *Acinetobacter* species are highlighted.
