# Supplementary material for: Molecular Characterization and Genetic Diversity of Haplogroup E Human Lice in Guinea, West Africa
Source: Microorganisms. 2021 Jan 27;9(2):257. doi: 10.3390/microorganisms9020257 (PMC7911403; doi:10.3390/microorganisms9020257)
Supplement: Supplementary file 1 [file microorganisms-09-00257-s001.pdf]

microorganisms

MDPI

Supplementary Materials: Molecular Characterization and Genetic Diversity of Haplogroup E Human Lice in Guinea, West Africa

Alissa Hammoud [1], Meriem Louni [2,3,*], Mamadou Cellou Baldé [4], Abdoul Habib Beavogui [5,6], Philippe Gautret [2], Didier Raoult [1], Florence Fenollar [2], Dorothée Misse [7] and Oleg Mediannikov [1,*]

Table S1. Table representing details about lice collected in Rural Maferenya and Urban Kindia, Guinea.

Host	Host Age	Nb of Lice	ID	Sex	Stage	Tube ID
			**Maférinyah**			
1	6	3	PGM1-1	M	A	1
			PGM1-2	F	A	2
			PGM1-3	F	A	3
2	9	1	PGM2-1	-	L3	4
3	8	3	PGM3-1	M	A	5
			PGM3-2	-	L3	6
			PGM3-3	M	A	7
4	8	3	PGM4-1	F	A	8
			PGM4-2	-	L3	9
			PGM4-3	-	L2	10
5	2	6	PGM5-1	-	L2	11
			PGM5-2	-	L2	12
			PGM5-3	M	A	13
			PGM5-4	M	A	14
			PGM5-5	-	L3	15
			PGM5-6	F	A	16
6	5	2	PGM6-1	-	L3	17
			PGM6-2	-	L3	18
7	6	4	PGM7-1	F	A	19
			PGM7-2	M	A	20
			PGM7-3	F	A	21
			PGM7-4	F	A	22
8	7	1	PGM8-1	M	A	23
9	34	5	PGM9-1	M	A	24
			PGM9-2	M	A	25
			PGM9-3	-	L2	26
			PGM9-4	-	L3	27
			PGM9-5	-	L3	28
10	8	1	PGM10-1	-	L3	29
11	9	5	PGM11-1	-	L2	30
			PGM11-2	-	L3	31
			PGM11-3	-	L3	32
			PGM11-4	-	L3	33
			PGM11-5	-	L3	34
12	11	4	PGM12-1	F	A	35
			PGM12-2	F	A	36
			PGM12-3	M	A	37
			PGM12-4	-	L2	38
13	4	3	PGM13-1	F	A	39
			PGM13-2	-	L3	40
			PGM13-3	-	L3	41
14	3	6	PGM14-1	-	L2	42
			PGM14-2	-	L3	43

			PGM14-3	-	L3	44
			PGM14-4	-	L3	45
			PGM14-5	-	L3	46
			PGM14-6	-	L3	47
15	6	6	PGM15-1	F	A	48
			PGM15-2	F	A	49
			PGM15-3	F	A	50
			PGM15-4	M	A	51
			PGM15-5	-	L3	52
			PGM15-6	-	L2	53
16	7	10	PGM16-1	-	L1	54
			PGM16-2	F	A	55
			PGM16-3	M	A	56
			PGM16-4	M	A	57
			PGM16-5	M	A	58
			PGM16-6	M	A	59
			PGM16-7	-	L3	60
			PGM16-8	-	L3	61
			PGM16-9	-	L3	62
			PGM16-10	-	L3	63
17	35	3	PGM17-1	-	L2	64
			PGM17-2	-	L2	65
			PGM17-3	-	L3	66
18	28	1	PGM18-1	F	A	67
19	6	4	PGM19-1	F	A	68
			PGM19-2	-	L3	69
			PGM19-3	-	L2	70
			PGM19-4	-	L2	71
20	12	1	PGM20-1	-	L3	72
21	9	4	PGM21-1	F	A	73
			PGM21-2	F	A	74
			PGM21-4	F	A	75
22	22	5	PGM22-1	F	A	76
			PGM22-2	F	A	77
			PGM22-3	-	L3	78
			PGM22-4	-	L3	79
			PGM22-5	-	L2	80
23	8	4	PGM23-1	F	A	81
			PGM23-2	F	A	82
			PGM23-3	F	A	83
			PGM23-4	M	A	84
24	9	13	PGM24-1	M	A	85
			PGM24-2	M	A	86
			PGM24-3	-	L2	87
			PGM24-4	-	L2	88
			PGM24-5	-	L2	89
			PGM24-6	-	L2	90
			PGM24-7	-	L3	91
			PGM24-8	-	L3	92
			PGM24-9	-	L3	93
			PGM24-10	-	L3	94
			PGM24-11	-	L3	95
			PGM24-12	-	L3	96
			PGM24-13	-	L3	97
25	4	3	PGM25-1	F	A	98
			PGM25-2	F	A	99
			PGM25-3	-	L3	100
26	6	1	PGM26-1	M	A	101

27	8	1	PGM27-1	F	A	102
28	9	2	PGM28-1	F	A	103
			PGM28-2	-	L3	104
29	12	3	PGM29-1	-	L3	105
			PGM29-2	-	L3	106
			PGM29-3	-	L3	107
30	7	1	PGM30-1	M	A	108
31	4	1	PGM31-1	F	A	109
32	3	3	PGM32-1	F	A	110
			PGM32-2	F	A	111
			PGM32-3	F	A	112
33	10	3	PGM33-1	F	A	113
			PGM33-2	F	A	114
			PGM33-3	F	A	115
34	10	2	PGM34-1	F	A	116
			PGM34-2	-	L3	117
35	9	3	PGM35-1	F	A	118
			PGM35-2	M	A	119
			PGM35-3	-	L3	120
36	8	4	PGM36-1	-	L3	121
			PGM36-2	-	L2	122
			PGM36-3	-	L1	123
			PGM36-4	-	L1	124
37	17	3	PGM37-1	F	A	125
			PGM37-2	F	A	126
			PGM37-3	M	A	127
38	13	3	PGM38-1	F	A	128
			PGM38-2	F	A	129
			PGM38-3	F	A	130
			**Kindia**			
1	8	1	PGK1-1	F	A	131
2	M-30	1	PGK2-1	-	L3	132
3	M-6	2	PGK3-1	M	A	133
			PGK3-2	-	L2	134
4	7	1	PGK4-1	-	L3	135
5	10	1	PGK5-1	M	A	136
6	8	1	PGK6-1	M	A	137
7	11	1	PGK7-1	-	L3	138
8	62	1	PGK8-1	F	A	139
9	6	1	PGK9-1	F	A	140
10	22	4	PGK10-1	-	L3	141
			PGK10-2	M	A	142
			PGK10-3	-	L3	143
			PGK10-4	F	A	144
11	9	11	PGK11-1	F	A	145
			PGK11-2	F	A	146
			PGK11-3	-	L3	147
			PGK11-4	M	A	148
			PGK11-5	M	A	149
			PGK11-6	F	A	150
			PGK11-7	M	A	151
			PGK11-8	F	A	152
			PGK11-9	M	A	153
			PGK11-10	F	A	154
			PGK11-11	-	L3	155

Table S2. Geographical and frequencies occurrences of *cytb* haplotypes of human head and body lice worldwide.

Haplotypes	Africa														Asia												Europe					Oceania			America													Total	Accession number	Head or Body
	Algeria	Burundi	Ethiopia	Kenya	Madagascar	Rwanda	Senegal	Egypt	South Africa	Mali	DR Congo	Congo Braz	Niger	Gabon	China	Iran	Japan	Laos	Mongolia	Nepal	Philippines	Russia	Taiwan	Yemen	Pakistan	Israel	France	Germany	Hungary	Portugal	United Kingdom	Australia	New Zelande	Papua-new-guinea	Colombia	Amazonia	Brazil	Canada	Ecuador	Honduras	Mexico	Panama	Peru	USA	Chille	Argentina	Shrunken head			
A1					1																																											1	KM579538	B
A2																						1																										1	KM579539	B
A3																																											1					1	KM579540	B
A4																																												4				4	KM579541	B
A5		18	21	7	17	13		91			28	24		11		2		1	1	45	5	27		1	9	7	95	4	10	4		38	10	17	2	1	10		1	3	1	1	9	34	1		1	570	KM579542	H & B
A6			3																																													3	KM579543	B
A7																																											1					1	KM579544	B
A8																						1						2																				3	KM579545	B
A9			1																																													1	KM579546	B
A10																				20																												20	KM579547	B
A11																	10																															10	KM579548	B
A12																																		1				7			8							16	KM579549	H & B
A13															6				5																													11	KM579550	H & B
A14																						1																										1	KM579551	H & B
A16																					9											9																18	KM579552	H
A17	2						72					64		306							1						5				4	10	6											8				478	KM579553	H & B
A18																																				2												2	KM579554	H
A19																							1																									1	KM579555	H
A45																				1																												1	KM579566	B
A55																										4																						4	KX232678	H
A56																										1																						1	KX232679	H
A57												5																																				5	KX444540	H
A58												4																																				4	KX444541	H
A59												1																																				1	KX444542	H
A60																											5																					5	MF672001	H
A61																											2																					2	MF672002	H
A62																											1																					1	MF672003	H
A63																																				9												9	MH429036	H
A64																																				2												2	MH429037	H
A65																																				3												3	MH429038	H
A66											7																																					7	MH230928	H
A67											2																																					2	MH230927	H
A68											2																																					2	MH230926	H
A69											1																																					1	MH230925	H
A70											1																1																					2	MN635432	H
A71																											1																					1	MN635433	H
A72														1																																		1	MN515375	H
A73														1																																		1	MN515376	H
A70-I											1																																					1	MH117941	H
A71-I											1																																					1	MH117942	H
A72-I											2																																					2	MH117943	B
A73-I											2																																					2	MH117944	H B
A74														1																																		1	MN515377	H
A75														1																																		1	MN515378	H
A76														1																																		1	MN515370	H
A77														1																																		1	MN515371	H
A78														2																																		2	MN515372	H
B31																											1																					1	KX249763	H
B32																																								1								1	KM579556	H
B33																																								5	1			16				22	KM579557	H
B34																																												2				2	KM579558	H
B36	24								59																	8	32	3			28									1				20	1			176	KM579559	H
B45																											1																					1	KX249764	H
B37																																								1								1	KX249776	H
B38																																								1								1	KX249777	H
B39																											4																					4	MF672004	H
B40																											2																					2	MN635434	H
F54																																				89												89	MH429039	H
F1																																				5												5	MH429040	H
F2																																				1												1	MH429041	H
F3																																				2												2	MH429042	H
F4																																				1												1	MH429043	H
F5																																				1												1	MH429044	H
F6																																				1												1	MH429045	H
F7																																				3												3	MH429046	H
C40			1																																													1	KM579561	H
C41																				17					79		2																					98	KM579562	H
C42			7																																													7	KM579563	H
C43			1																																													1	KM579564	H
C44			51																																													51	KM579565	H
C51																											1																					1	KX249765	H
C45																									1																							1	KX249778	H
C74												32		13																																		45	KX444547	H
C75												6		4																																		10	KX444548	H
C76												3																																				3	KX444549	H
C77												2																																				2	KX444550	H
C78												1																																				1	KX444551	H
C79												1																																				1	KX444552	H
C80														1																																			MN515373	H
E39							39			14																																						53	KM579560	H
E46										22				1																																		23	KX249780	H
E47										15																																						15	KX249781	H
E48							1			15																																						16	KY937987	H
E49										2																																						2	KY937988	H
E50										1																																						1	KY937989	H
E51										1																																						1	KY937990	H
E52													3																																			3	MH429047	H
E53													32																																			32	MH429048	H
E54													1																																			1	MH429049	H
E55													1																																			1	MH429050	H
E56																											9																					9	MF672005	H
E57																											3																					3	MF672006	H
E58																											1																					1	MF672007	H
E59																											7																					7	MF672008	H
E60																											1																					1	MF672009	H
E61																											1																					1	MF672010	H
E62											10																																					10	MH230921	H
E63																											4																					4	MG759552	H
E64																											2																					2	MG759553	H
E65																											1																					1	MG759554	H
E66																											1																					1	MG759555	H
E67																											1																					1	MG759556	H
																																																0		H
D60											5	3																																				8	KX249766	B
D61											2																																					2	KX249767	B
D62											2																																					2	KX249768	B
D63											3																																					3	KX249769	H
D64											2																																					2	KX249770	H
D65											12	11																																				23	KX249771	H
D66											4																																					4	KX249772	H
D67											20																																					20	KX249773	H B
D68			1																																													1	KX249779	H
D69			3																																													3	KX249774	B
D70			1																																													1	KX249775	H
D71												4																																				4	KX444544	H
D72												1																																				1	KX444545	H
D73												2																																				2	KX444546	H
D74												4																																				4	MH230924	H
D75												1																																				1	MH230923	H
D76												8																																				8	MH230922	H
D77											1																																					1	MH117945	B
D78											1																																					1	MH117946	H
D79											1																																					1	MH117947	B
D80											1																																					1	MH117948	B
D81											1																																					1	MH117949	H
D82											1																																					1	MH117950	H
D83											1																																					1	MH117951	B
D84											1																																					1	MH117952	B
D85											1																																					1	MH117953	H
	26	18	90	7	18	13	112	91	59	70	##	177	37	344	6	2	10	1	6	83	15	30	1	1	89	20	184	9	10	4	32	57	16	18	2	120	10	7	1	12	10	1	11	84	2	0	1	2023		

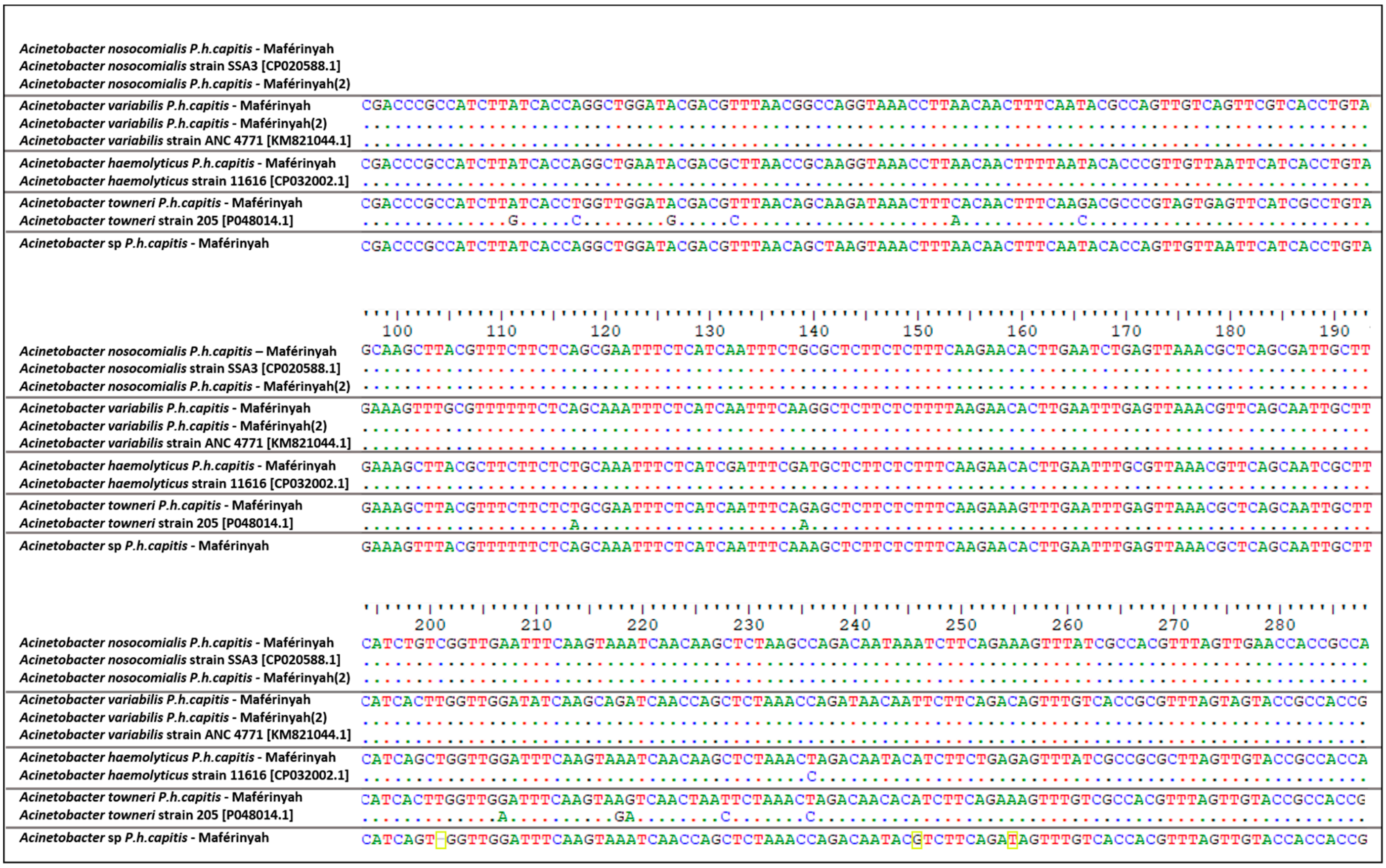

Figure S1. Alignment of a 288-bp fragment of the rpoB gene of Acinetobacter nosocomialis strain SSA3 [CP020588.1], Acinetobacter variabilis strain ANC 4771 [KM821044.1], Acinetobacter haemolyticus strain 11616 [CP032002.1] and Acinetobacter towneri strain 205 [P048014.1] with the five Acinetobacter species described in this study using BioEdit software. Mutations of our potential new specie 'Acinetobacter sp P.h.capitis – Maferenya' regarding other Acinetobacter species are highlighted.
